# Acetylcholinesterase-Inhibition and Antibacterial Activity of *Mondia whitei* Adventitious Roots and *Ex vitro*-Grown Somatic Embryogenic-Biomass

**DOI:** 10.3389/fphar.2016.00335

**Published:** 2016-10-03

**Authors:** Ponnusamy Baskaran, Aloka Kumari, Bhekumthetho Ncube, Johannes Van Staden

**Affiliations:** Research Centre for Plant Growth and Development, School of Life Sciences, University of KwaZulu-NatalPietermaritzburg, South Africa

**Keywords:** acetylcholinesterase-inhibition, adventitious root, bioactivity, medicinal plant, somatic embryogenesis

## Abstract

*Mondia whitei* (Hook.f.) Skeels is an important endangered medicinal and commercial plant in South Africa. *In vitro* propagation systems are required for biomass production and bioactivity analysis to supplement wild resources/stocks. Adventitious roots from somatic embryogenic explants using suspension culture and *ex vitro*-grown plants produced via somatic embryogenesis were established using different plant growth regulator treatments. The adventitious root biomass and different parts of *ex vitro*-grown and mother plants were used to investigate the potential for acetylcholinesterase (AChE) and antibacterial activities. Adventitious roots derived from 2.5 μM indole-3-acetic acid (IAA) treatments and *ex vitro-*grown plants derived from *meta*-topolin riboside and IAA treatments gave the best AChE and antibacterial activities. The *in vitro-*established *M. whitei* and *ex vitro* biomass have comparable ability to function as inhibitors of acetylcholinesterase and antibacterial agents, and can be used as potent bioresources in traditional medicine.

## Introduction

*Mondia whitei* (Hook.f.) Skeels is an important endangered medicinal plant included in the Red Data List of South African plants as a result of its over-exploitation for various medicinal uses (SANBI, 2013; Baskaran et al., 2015; Van Wyk, 2015). The conservation status and medicinal importance requires the development of an efficient mass propagation system. The plant’s root is used in the treatment of various diseases including those related to nervous conditions (Gelfand et al., 1985; Noumi et al., 1998; Oketch-Rabah, 2012; Baskaran et al., 2015; Van Wyk, 2015). Accordingly, assay of acetylcholinesterase (AChE: primary cholinesterase enzyme for nerve agents) and antibacterial properties for numerous clinical problems caused by bacteria needs to be examined in *M. whitei.* The *in vitro* established micropropagated plants requires evaluation for bioactivity to ascertain if the medicinal properties remains unchanged (Nagarajan et al., 2011; García-Pérez et al., 2012; Baskaran et al., 2014). Many bacterial strains (for example, *Enterococcus faecalis*) are becoming resistant to antibiotics; therefore, the search for potent medicinal plants represent a natural source that requires exploration. Furthermore, the application of biotechnological techniques has permitted harvesting and evaluation of *in vitro* regenerated plants and plant materials at various stages of plant growth and development (Nagarajan et al., 2011; García-Pérez et al., 2012; El-Bakry et al., 2014). A report of *in vitro* adventitious root culture and *ex vitro*-grown plants derived from somatic embryogenesis are required to test the bioactivity to support and utilization in medicinal systems as well as to preserve existing *M. whitei* populations. Establishing adventitious roots by suspension culture and *ex vitro*-grown plants from somatic embryogenetic origin would accelerate large-scale biomass and conservation in addition to supplementing pharmaceutical products to study the biological significance of the species bioactivity (Cui et al., 2010; Ho et al., 2013; Baskaran et al., 2014).

*In vitro* culture developed plant biomass has found pharmacological applications worldwide (García-Pérez et al., 2012; Ho et al., 2013). Acetylcholinesterase (AChE: primary cholinesterase enzyme) functions as a neurotransmitter and the assay of AChE activity plays an important role in diagnostic, nerve agents, *in vitro* characterization of toxins and drugs, including potential treatments for Alzheimer’s disease (Pohanka et al., 2011). Numerous bacterial diseases, including diarrhea, can be alleviated by extracts from various parts of *in vitro* and *ex vitro*-grown plants (Nagarajan et al., 2011; García-Pérez et al., 2012). The aim of this study therefore, was to develop a simple and efficient protocol for the production of adventitious roots using somatic embryogenic explants in suspension culture and to examine the AChE and antibacterial activities from the biomass of the adventitious roots and different parts of *ex vitro*-grown plants derived via somatic embryogenesis, using the mother plant for comparison. The hormonal effects of the adventitious root and somatic embryogenesis-derived *ex vitro* plants of *M. whitei* biomass on AChE-inhibition and antibacterial activities were also determined.

## Materials and Methods

### Production of Adventitious Roots in Suspension Culture

Torpedo stage somatic embryos (SEs) of *M. whitei* were collected using a previously described somatic embryogenesis protocol (Baskaran et al., 2015) on solid (8 g L^-1^ agar) MS (Murashige and Skoog, 1962) medium with 40 g L^-1^ sucrose, 20 μM 2,4-D, and 1 μM TDZ. The embryos (approximately 7 – 10) were transferred to 250 mL Erlenmeyer flasks containing 50 mL liquid MS medium with 30 g L^-1^ sucrose for induction of roots for 4 weeks. The roots (1 g L^-1^, 3–5 cm long) were transferred to 50 mL liquid MS medium containing 30 g L^-1^ sucrose plus 2.5 – 10 μM indole-3-acetic acid (IAA) or indole-3-butyric acid (IBA) and 2.5 – 15 μM naphthaleneacetic acid (NAA) in a 250 mL Erlenmeyer flask to optimize a concentration of auxin for enhancement of adventitious root biomass for 8 weeks. The liquid cultures were maintained on an orbital shaker at 100 rpm at 25 ± 2°C and light intensity of 40 μmol m^-2^ s^-1^ provided by cool white fluorescent light (OSRAM L 58 W/740, South Africa) with a 16 h photoperiod. All experiments were conducted three times with five replicates per treatment. In all experiments, medium lacking plant growth regulators served as controls. The chemicals used were of analytical grade (Biolab, South Africa; Oxoid, England and Sigma, USA). All media were adjusted to pH 5.8 with 0.1 N NaOH and/or 0.1 N HCl and autoclaved at 121°C for 20 min.

Roots were filtered through sieves (200 μm), and the fresh weight (FW) measured after rinsing once with sterile distilled water and blotting away the surface water. The roots were dried at 50°C for 1 day, and the root dry weight (DW) was recorded. Adventitious root growth ratio was calculated as harvested DW (g) - inoculated DW (g)/inoculated DW (g). The data were statistically analyzed for analysis of variance (ANOVA), and are presented as mean ± standard error of three independent experiments. Treatment means were separated using Duncan’s multiple range test at 5% probability level. All statistical analysis was done using SPSS for Windows version 23 (SPSS Inc., Chicago, IL, USA).

### *Ex vitro* Plants from Somatic Embryogenesis

The 18-month-old somatic embryogenesis system produced (Baskaran et al., 2015) *ex vitro-*grown plants of *M. whitei* derived from different treatments [50 g L^-1^ sucrose + 20 μM 2,4-dichlorophenoxy acetic acid (2,4-D), 40 g L^-1^ sucrose + 20 μM 2,4-D + 1 μM thidiazuron (TDZ), 35 g L^-1^ sucrose + 20 μM picloram + 1 μM benzyladenine (BA), 20 g L^-1^ sucrose + 0.5 μM *meta*-topolin riboside (*m*TR) + 0.25 μM IAA and 20 g L^-1^ sucrose + 0.5 μM kinetin (Kin) + 0.25 μM IAA; **Tables 1** and **2**] were used for the bioassay studies.

**Table 1:** Acetylcholinesterase-inhibition in adventitious roots and various plant parts of *ex vitro* and garden-grown mother plants of *Mondia whitei.*

Sucrose (g L^-1^) + PGR treatment (μM)	Source of plant material	Plant part	AChE-inhibition IC_50_ (mg ml^-1^)
Control 30 + 2.5 IAA 30 + 10 IAA 30 + 2.5 IBA 30 + 10 IBA 30 + 2.5 NAA 30 + 10 NAA 30 + 15 NAA 50 + 20 2,4-D 40 + 20 2,4-D + 1 TDZ 35 + 20 picloram + 1 BA 20 + 0.5 *m*TR + 0.25 IAA 20 + 0.5 Kin + 0.25 IAA	*In vitro* culture *Ex vitro* plant Mother plant	Adventitious root Root Leaf Stem Root Leaf Stem Root Leaf Stem Root Leaf Stem Root Leaf Stem Root Leaf Stem	2.13 ± 0.21 ef 1.33 ± 0.14 b 3.60 ± 0.18 hi 3.57 ± 0.46 hi 2.57 ± 0.15 fg 3.44 ± 0.37 hi 3.35 ± 0.17 hi 3.91 ± 0.20 i **0.81 ± 0.14** a 2.96 ± 0.20 gh 2.00 ± 0.12 de 2.29 ± 0.18 ef **0.66 ± 0.25** a 1.72 ± 0.26 cd 2.13 ± 0.29 ef 2.13 ± 0.29 ef 3.90 ± 0.22 i 1.14 ± 0.15 b **0.88 ± 0.17** a **0.65 ± 0.09** a 1.05 ± 0.24 b **0.88 ± 0.10** a 1.67 ± 0.21 cd 1.30 ± 0.17 b 2.61 ± 0.22 fg 1.32 ± 0.60 b

*PGR, Plant growth regulator. IC_*50*_ for galantamine (positive control) = 1.56 ± 0.18 μM. Values in bold font are considered significantly higher AChE-inhibitory activity. Results are mean ± standard error (*n* = 3). Means followed by same letters in each column are not significantly different (*P =* 0.05) using Duncan’s multiple range test.*

**Table 2:** Antibacterial activity of adventitious roots and various plant parts of *ex vitro* and garden-grown mother plants of *M. whitei.*

Sucrose (g L^-1^) + PGR treatment (μM)	Source of plant material	Plant part	Minimum inhibitory concentration (mg ml^-1^)					
			*B.s ^+ve^*	*E.f ^+ve^*	*M.l ^+ve^*	*S.a ^+ve^*	*E.c ^-ve^*	*K.p ^-ve^*
Control 30 + 2.5 IAA 30 + 10 IAA 30 + 2.5 IBA 30 + 10 IBA 30 + 2.5 NAA 30 + 10 NAA 30 + 15 NAA 50 + 20 2,4-D 40 + 20 2,4-D + 1 TDZ 35 + 20 Pic + 1 BA 20 + 0.5 *m*TR + 0.25 IAA 20 + 0.5 Kin + 0.25 IAA Neomycin (positive control)	*In vitro* culture *Ex vitro* plant Mother plant	Adventitious root Root Leaf Stem Root Leaf Stem Root Leaf Stem Root Leaf Stem Root Leaf Stem Root Leaf Stem –	**0.39** **0.195** **0.39** **0.39** **0.195** 3.125 **0.39** **0.39** **0.098** 1.56 **0.098** **0.098** 1.56 **0.098** **0.098** 1.56 **0.098** **0.39** **0.195** **0.098** **0.098** 1.56 **0.098** **0.098** **0.39** **0.098** 0.024	3.125 3.125 1.56 1.56 1.56 3.125 3.125 1.56 1.56 **0.195** **0.195** 1.56 **0.098** **0.195** **0.195** **0.098** **0.098** **0.39** **0.098** **0.098** 1.56 **0.098** **0.195** 3.125 0.78 **0.098** 0.012	1.56 **0.39** **0.39** **0.39** **0.39** 3.125 1.56 1.56 3.125 3.125 3.125 3.125 3.125 3.125 3.125 3.125 1.56 1.56 3.125 3.125 3.125 0.78 3.125 1.56 0.78 3.125 0.0061	3.125 1.56 1.56 1.56 1.56 3.125 1.56 1.56 1.56 6.25 6.25 12.5 12.5 12.5 12.5 12.5 12.5 12.5 12.5 6.25 6.25 12.5 12.5 6.25 6.25 3.125 0.0061	3.125 1.56 1.56 3.125 1.56 3.125 1.56 **0.39** 3.125 3.125 3.125 6.25 3.125 6.25 6.25 6.25 6.25 1.56 3.125 3.125 3.125 1.56 1.56 3.125 0.78 3.125 0.0015	0.78 **0.39** **0.39** 1.56 1.56 1.56 1.56 **0.39** 1.56 3.125 3.125 6.25 6.25 3.125 3.125 3.125 3.125 1.56 1.56 1.56 3.125 1.56 1.56 6.25 0.78 3.125 0.0061

*Extracts with values in bold font are considered very active (MIC < 1 mg ml^-*1*^). Results are mean ± standard error (*n* = 3). PGRs, Plant growth regulators. MIC (mg ml^-*1*^); B.s, Bacillus subtilis; E.f, Enterococcus faecalis; M.l, Micrococcus luteus; S.a, Staphylococcus aureus; E.c, Escherichia coli; K.p, Klebsiella pneumoniae.*

Different plant materials (root, leaf, and stem) were harvested from *ex vitro*-grown [1:1 (v/v) vermiculite:soil mixture under greenhouse conditions (25 ± 2°C under natural photoperiod conditions and a midday PPF of 950 ± 50 μmol m^-2^ s^-1^)] and mother plant (15-years-old) at the same time period (to avoid seasonal differences), washed thoroughly with sterile water and dried in an oven set at 50°C in the dark.

### Bioactivity Studies

**FIGURE 1:**
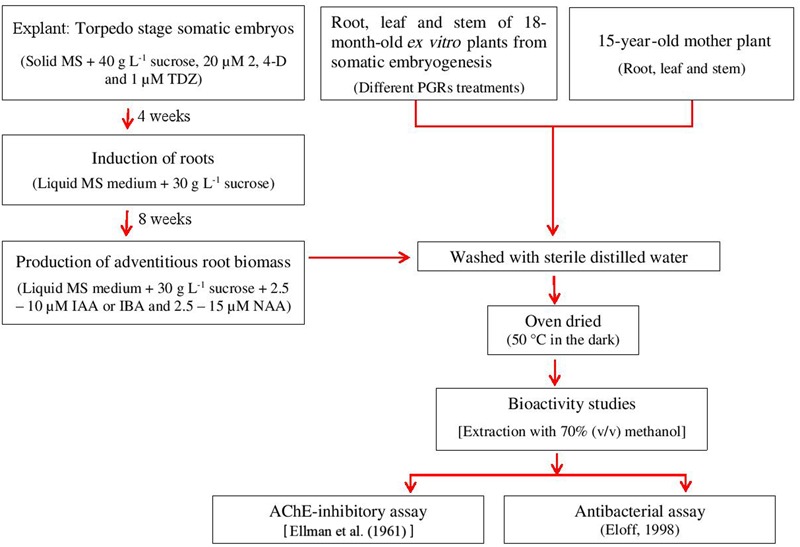
**A schematic diagram for bioactivity from adventitious roots using liquid cultures and *ex vitro*-grown somatic embryos derived plants and mother plant of *M. whitei***.

The oven-dried plant materials (*ex vitro*-grown, mother plant and adventitious root: 1 g powder each) were extracted with 70% (v/v) methanol (100 mL) using a sonication bath for 1 h. The extracts were filtered through a Büchner funnel using Whatman no. 1 filter paper and the solvent evaporated under reduced pressure at 30°C. Dried extracts were kept at 4°C until use. Each extract was tested for acetylcholinesterase (AChE) inhibitory and antibacterial properties. The bioactivity studies from *in vitro, ex vitro*, and mother plant materials of *M. whitei* are described by a schematic diagram (**Figure 1**).

#### AChE-Inhibitory Assay

The colorimetric method of Ellman et al. (1961) using a 96-well microplate as described by Fawole et al. (2010) was used. Each sample was evaluated in triplicate. Galantamine was used as a positive control. Following the calculation of the rate of reaction for each sample, galantamine or blank, the percentage inhibition was calculated using the formula: AChE inhibition (%) = [1 – (sample reaction rate/blank reaction rate)] × 100. A non-linear regression analysis was done using GraphPad Prism (version 4.03) software for the determination of the IC_50_ values. Data were subjected to analysis of variance (SPSS, version 23.0), and significant mean values were further separated using Duncan’s multiple range test.

#### Antibacterial Assay

The microtiter bioassay (Eloff, 1998) was used to determine antibacterial activity. Extracts were tested against Gram-positive bacteria (*Bacillus subtilis* [ATCC 6051], *E. faecalis* [ATCC 19433], *Micrococcus luteus* [ATCC 4698], and *Staphylococcus aureus* [ATCC 12600]) and Gram-negative bacteria (*Escherichia coli* [ATCC 11775] and *Klebsiella pneumoniae* [ATCC 13883]). A positive control, neomycin (μg ml^-1^ in the first well) was used against each bacterial strain. Each determination was done in duplicate, and the assay was repeated twice.

## Results and Discussion

### Production of Adventitious Roots

**FIGURE 2:**
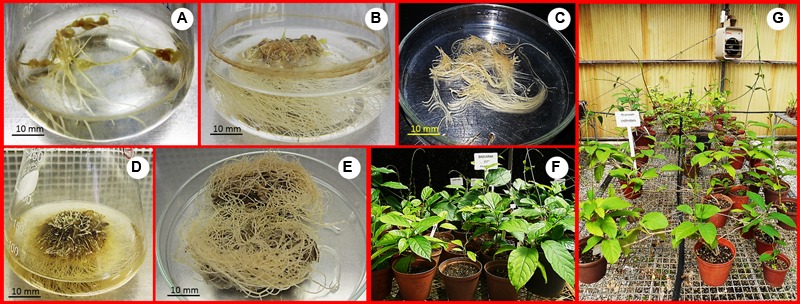
**Production of adventitious roots using liquid cultures and *ex vitro*-grown somatic embryos derived plants of *M. whitei*. (A)** Root induction from torpedo-stage somatic embryo explants after 2 weeks culture. **(B)** Root induction after 4 weeks culture. **(C)** Root explants for adventitious root culture. **(D)** Production of adventitious roots in 10 μM IAA after 8 weeks culture. **(E)** Adventitious roots in 10 μM NAA. **(F)** Establishment of *ex vitro*-grown plants in the greenhouse. **(G)** *Ex vitro*-grown plants after 18 months.

**FIGURE 3:**
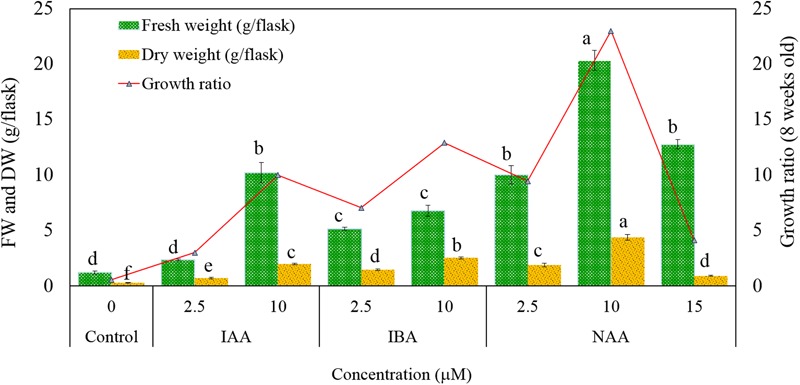
**Different concentrations of auxins for adventitious root growth [fresh weight (FW) and dry weight (DW)] and growth ratio of *M. whitei*.** Results are mean ± standard error (*n* = 3). The bar containing different letters in graph indicates significant differences at *P =* 0.05 of significance using Duncan’s multiple range test.

Root induction was achieved using the torpedo-stage somatic embryo explants in liquid MS medium containing 30 g L^-1^ sucrose after 4 weeks (**Figures 2A,B**). The adventitious roots were established from root explants cultured in liquid MS medium containing 30 g L^-1^ sucrose and various concentrations of auxins (IAA, IBA, and NAA; **Figure 2C**). Normal adventitious roots form, but require auxins *in vitro* (Baskaran and Jayabalan, 2009; Cui et al., 2010; El-Bakry et al., 2014; Steffens and Rusmussen, 2016). Increased concentrations (>2.5 μM) of auxins improved biomass production (>6.79 g/flask FW and >2.00 g/flask DW; **Figures 2D** and **3**), and the highest production of biomass (20.34 g/flask FW and 4.37 g/flask DW) as well as a good growth ratio was observed on medium containing 10 μM NAA (**Figures 2E** and **3**). The biomass production was markedly suppressed with an increased concentration (15 μM) of NAA (**Figure 3**). High auxin levels are often suppressors for adventitious root growth and production of biomass (Baskaran and Jayabalan, 2009; Cui et al., 2010). In this study, the growth ratio was lower in control, 2.5 μM IAA and 15 μM NAA (**Figure 3**). These results indicate that the type of auxin and its concentration is important for growth of adventitious roots and biomass production and were in accordance with other previous reports (Baskaran and Jayabalan, 2009; El-Bakry et al., 2014).

### AChE-Inhibitory and Antibacterial Activities

In this study, AChE-inhibitory activity was observed in adventitious roots, different plant parts of *ex vitro*-grown plants (**Figures 2F,G**) and the mother plant of *M. whitei* (**Table 1**). The root and stem of mother plant exhibited significant AChE-inhibitory activity, but this did not differ significantly with those of the adventitious roots (2.5 μM IAA) and roots of *ex vitro-*grown plants from 20 g L^-1^ sucrose + 0.5 μM *m*TR + 0.25 μM IAA and 20 g L^-1^ sucrose + 0.5 μM Kin + 0.25 μM IAA treatments, respectively (**Table 1**). The IC_50_ values recorded in extracts of adventitious roots derived from control, IAA (10 μM), IBA, and NAA treatments and roots, leaves and stems of *ex vitro*-grown plants derived from 35 g L^-1^ sucrose + 20 μM picloram + 1 μM BA treatments did not exhibit good AChE-inhibitory activity (**Table 1**). The inhibitory activity was significantly higher in roots (50 + 20 2,4-D), leaves (40 g L^-1^ sucrose + 20 2,4-D + 1 TDZ; 20 g L^-1^ sucrose + 0.5 *m*TR + 0.25 IAA; 20 g L^-1^ sucrose + 0.5 Kin + 0.25 IAA) and stems (20 g L^-1^ sucrose + 0.5 *m*TR + 0.25 IAA) of *ex vitro* plants (**Table 1**). Overall, AChE-inhibitory activity was good in all parts of *ex vitro*-grown plants derived from 20 g L^-1^ sucrose + 0.5 *m*TR + 0.25 IAA treatments (**Table 1**). These results indicate that the AChE-inhibitory activity differs within the treatments and plant parts. The superior inhibitory effects demonstrated by IAA and *m*TR treatments suggest that they may be regulating certain secondary compounds during *in vitro* and *ex vitro* processes. Moreover, adventitious roots and *ex vitro*-grown plants treated with specific PGRs in different concentrations could be an efficient substitute for improving wild populations. In addition, the therapeutic potential of *ex vitro* plants may influence somatic embryogenic phases. *Ex vitro*-grown and *in vitro* regenerated plants with PGR treatments have been reported with potential bioactivity in other plant species (Baskaran et al., 2014).

Antibacterial activity from adventitious roots and different parts of *ex vitro* and mother plants of *M. whitei* showed antibacterial activity against tested human pathogenic bacteria (**Table 2**). The bioactivity test in *in vitro* established tissues has been reported to support plants used in traditional medicine and prevent loss of wild populations (Baskaran et al., 2014). Extracts of adventitious roots showed good activity against both Gram-positive (*B. subtilis* and *M. luteus*) and Gram-negative (*E. coli* and *K. pneumoniae*) bacteria; however, no bioactivity was observed for adventitious roots against Gram-positive (*E. faecalis* and *S. aureus*) bacteria (**Table 2**). Active secondary metabolism has been reported in adventitious roots of ginseng (Yu et al., 2000; Hahn et al., 2003). The higher bioactivity was recorded in extracts of different parts of *ex vitro*-grown plants derived from different treatments and were more potent against *B. subtilis* and *E. faecalis* bacteria (**Table 2**). On the other hand, none of the extracts of the *ex vitro-*grown and mother plants exhibited activity against both Gram-positive (*M. luteus* and *S. aureus*) and Gram-negative (*E. coli* and *K. pneumoniae*) bacteria (**Table 2**). The results of this study indicate that the antibacterial activity depended on the source of plant material, tissue type and concentration of PGRs in the treatments and are in accordance with results obtained with *Agapanthus praecox* (Baskaran et al., 2014). Accumulation of secondary compounds as regulated by PGRs during *in vitro* and *ex vitro*-growth processes may have affected the bioactivity. However, the biochemical mechanisms surrounding these still needs to be investigated in the plant species. The results of the present study suggest that *in vitro* and *ex vitro*-grown plant parts as obtained with specific PGR treatments can effectively be used for the treatment of bacterial ailments in traditional medicine.

## Conclusion

Pharmacological investigations were conducted to confirm acetylcholinesterase (AChE) and antibacterial activities of *in vitro* adventitious roots and *ex vitro*-acclimatized plants of *M. whitei*. The bioactivity was found to vary with specific concentration and combinations of PGRs during the *in vitro* phases. Established *in vitro* plants had stronger bioactivity and ability to function as inhibitors of acetylcholinesterase and antibacterial agents and thus offer potential for use as potent bioresources in traditional medicine and other commercial applications.

## Author Contributions

PB and JVS contributed in perceiving and designing the study. PB, AK, and BN equally contributed with experiments and collection of data for the study. Data analysis and draft of the manuscript were completed by all authors. All the authors approved the content of the manuscript.

## Conflict of Interest Statement

The authors declare that the research was conducted in the absence of any commercial or financial relationships that could be construed as a potential conflict of interest.
